# 6-[(*tert*-Butyl­dimethyl­sil­yl)­oxy]-3-ethenyl-7-meth­oxy-4-[(tri­methyl­sil­yl)ethyn­yl]naphtho­[2,3-*c*]furan-1(3*H*)-one

**DOI:** 10.1107/S2414314620002242

**Published:** 2020-02-21

**Authors:** Matthias Weil, Thomas Kremsmayr, Marko D. Mihovilovic

**Affiliations:** a Institute for Chemical Technologies and Analytics, Division of Structural Chemistry, TU Wien, Getreidemarkt 9/164-SC, A-1060 Vienna, Austria; b Institute of Applied Synthetic Chemistry, TU Wien, Getreidemarkt 9/163-OC, A-1060 Vienna, Austria; Goethe-Universität Frankfurt, Germany

**Keywords:** crystal structure, notoincisol, ethynyl group

## Abstract

The tricyclic core in the title compound, C_26_H_34_O_4_Si_2_, shows disorder of the furan ring and deviates slightly from planarity, with the largest displacement from the least-squares plane [0.166 (2) Å] for the major disordered part of the methine C atom. To this C atom the likewise disordered vinyl group is attached, lying nearly perpendicular to the tricyclic core. In the crystal, mutual C—H⋯π inter­actions between the methine group of the furan ring and the central ring of the tricyclic core of an adjacent mol­ecule lead to inversion-related dimers.

## Abstract 2

In the mol­ecule of the title compound, the tricyclic core, consisting of a naphthalene entity fused together with a furan ring, deviates slightly from planarity.

## Graphical abstract

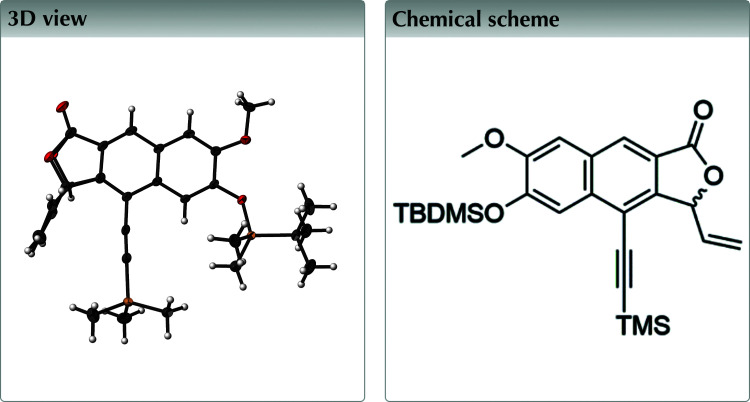

## Structure description

**Figure 1:**
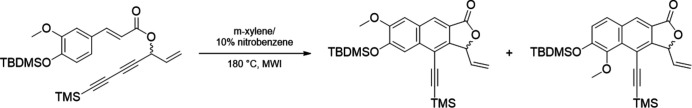
Reaction scheme to afford the title compound along with its regioisomer.

Notoincisol B is a naturally occurring polyenyne-hybrid compound, which was found to act as a promising partial agonist at the nuclear receptor PPARγ (Liu *et al.*, 2014 ▸). Attempting to make this novel scaffold synthetically accessible, a biomimetic pathway towards notoincisol B has been investigated (Kremsmayr, 2017 ▸). Within the proposed synthetic route, the lactone analogue of the characteristic tricyclic notoincisol B core structure was obtained *via* an intra­molecular de­hydro-inverse-electron-demand Diels–Alder-type cyclization reaction between a styrene and an alkyne moiety. Cyclization of a simplified notoincisol B model precursor substrate afforded the title compound, along with equal qu­anti­ties of a regioisomer and proved the synthetic feasibility of this key-step reaction (Fig. 1 ▸).

**Figure 2:**
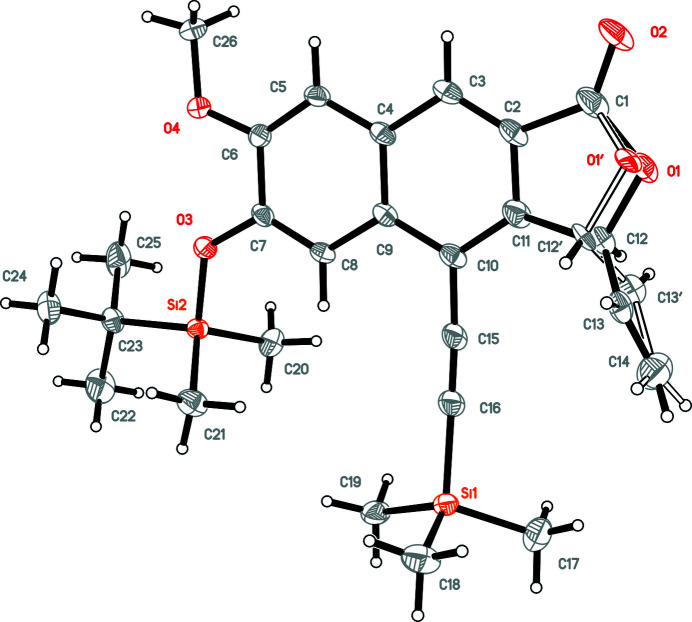
The mol­ecular structure of the title compound, showing anisotropic displacement ellipsoids at the 50% probability level. The disordered part with a minor contribution is shown with open bonds.

**Figure 3:**
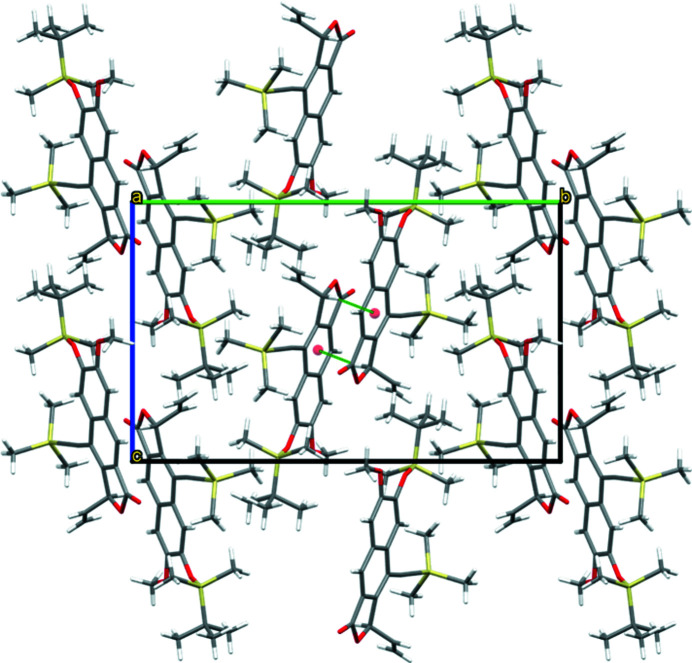
View of the crystal packing along [100]. Mutual C—H⋯π inter­actions (green lines) lead to the formation of inversion-related dimers, as emphasized in the middle of the unit cell. For clarity, only the disordered part with major contribution is shown.

The mol­ecular structure of the title compound is displayed in Fig. 2 ▸. A naphthalene entity to which a furan ring is fused makes up the tricyclic core of the mol­ecule, consisting of twelve C atoms (C1–C12) and one O atom (O1). Parts of the furan ring (O1, C12) and the attached vinyl group (C13, C14) are disordered over two sets of sites. The tricyclic core is non-planar, with an r.m.s. deviation of fitted atoms from the least-squares plane of 0.0778 Å. The highest deviation is 0.166 (2) Å for C12 (considering the major disordered part), which is also the atom to which the vinyl group (—C13=C14) is attached. The latter is nearly perpendicular to the tricyclic core, with a dihedral angle of 85.2 (2)° between the two moieties. The angle between the C10 atom of the tricyclic core and the attached ethynyl group (—C15≡C16—) is slightly bent [175.98 (16)°], just like the angle between the ethynyl group and the Si1 atom of the tri­methyl­silyl (TMS) group [178.46 (16)°]. Fig. 3 ▸ shows the packing of individual mol­ecules in the crystal. The bulky *tert*-(butyl­dimethyl­sil­yl)­oxy (TBDMSO) and tri­methyl­silyl (TMS) groups prevent π–π stacking, and the only remarkable inter­molecular inter­action between two adjacent mol­ecules are mutual weak C—H⋯π contacts. This involves the methine group (C12—H12) of the furan ring and the centroid of the central ring (C2,–C11; *Cg*1) of the tricyclic core [C12—H12⋯*Cg*1(1 − *x*, 1 − *y*, 1 − *z*); H12⋯*Cg*1 = 2.63 Å, C12⋯*Cg*1 = 3.622 (3) Å, C12—H12⋯*Cg*1 = 171°]. In this way, inversion-related dimers are formed (Fig. 3 ▸).

## Synthesis and crystallization

The synthesis followed a reported procedure (Kocsis & Brummond, 2014 ▸). A 20 ml microwave vial, equipped with a stirring bar, was charged with 7-(tri­methyl­sil­yl)hepta-1-en-4,6-diyn-3-yl (*E*)-3-{4-[(*tert*-butyl­dimethyl­sil­yl)­oxy]-3-meth­oxy­phen­yl}acrylate (0.400 g, 0.85 mmol, 1.00 equiv.), *m*-xylene (12.6 ml) and PhNO_2_ (1.4 ml 10% (*v*/*v*%) in *m*-xylene, final molarity *c* = 0.06 *M*). The vial was sealed and heated *via* microwave irradiation to 453 K for 15 min, resulting in a colour change from a light-green to a dark-brown solution (reaction progress was checked with TLC, Lp: EtOAc = 10:1). Upon completion, the crude mixture was transferred into a flask and solvents were evaporated under high vacuum at 333–343 K. The two resulting regioisomers were separated *via* flash chromatography (180 g SiO_2_, flow rate 50 ml min^−1^, using gradient Lp to Lp: EtOAc = 10:1 in 30 min, then 10:1 isocratically 10 min), affording 0.160 g (40%) of the title compound as a beige solid and 0.151 g (38%) of its regioisomer as a yellow oil. The title compound was recrystallized from ligroin, affording colourless material.

^1^H NMR (400 MHz, CDCl_3_). δ = 0.27 (*s*, 6H, TBDMS [–Si(CH_3_)_2_]}, 0.32 (*s*, 9H, TMS [–Si(CH_3_)_3_]}, 1.05 {*s*, 9H, TBDMS [–SiC(CH_3_)_3_]}, 3.97 (*s*, 3H, –OCH_3_), 5.37–5.51 (*m*, 1H, H1_*cis*_), 5.59–5.77 (*m*, 1H, H1_*trans*_), 5.92–6.12 (*m*, 2H, H2, H3), 7.23 (*s*, 1H, H_Ar_), 7.80 (*s*, 1H, H_Ar_), 8.23 (*s*, 1H, H_Ar_) p.p.m.

^13^C NMR (150 MHz, CDCl_3_). δ = −4.44 [*q*, TBDMS (–SiCH_3_)], −4.43 [*q*, TBDMS [–SiCH_3_)], 0.0 {q, TMS [–Si(CH_3_)_3_]}, 18.8 {*s*, TBDMS [–SiC(CH_3_)_3_]}, 25.8 {*q*, TBDMS [–SiC(CH_3_)_3_]}, 55.8 (*q*, –OCH_3_), 82.2 (*d*, C3), 98.5 (*s*, C_Ar_ or C_alkyne_), 106.8 (*s*, C_Ar_ or C_alkyne_), 108.3 (*d*, C_Ar_), 113.5 (*s*, C_Ar_ or C_alkyne_), 114.6 (*d*, C_Ar_), 119.8 (*t*, C1), 121.5 (*s*, C_Ar_), 125.3 (*d*, C_Ar_), 129.9 (*s*, C_Ar_), 132.0 (*d*, C2), 133.5 (*s*, C_Ar_), 143.3 (*s*, C_Ar_), 149.7 (*s*, C_Ar_), 152.9 (*s*, C_Ar_), 170.3 (*s*, C9′) p.p.m.

## Refinement

Crystal data, data collection and structure refinement details are summarized in Table 1 ▸. Parts of the furan ring (O1, C12) and the attached vinyl group (C13, C14) are disordered over two sets of sites, with a refined occupancy ratio of 0.793 (5):0.207 (5). The disordered part with the minor contribution is assigned a prime character.

## Supplementary Material

Crystal structure: contains datablock(s) global, I. DOI: 10.1107/S2414314620002242/bt4089sup1.cif

Structure factors: contains datablock(s) I. DOI: 10.1107/S2414314620002242/bt4089Isup2.hkl

CCDC reference: 1984687

Additional supporting information:  crystallographic information; 3D view; checkCIF report

## Figures and Tables

**Table 1:** Experimental details

Crystal data	
Chemical formula	C_26_H_34_O_4_Si_2_
*M*_r_	466.71
Crystal system, space group	Monoclinic, *P*2_1_/*c*
Temperature (K)	100
*a*, *b*, *c* (Å)	10.2628 (6), 20.5653 (13), 13.3923 (8)
β (°)	111.6378 (15)
*V* (Å^3^)	2627.4 (3)
*Z*	4
Radiation type	Mo *K*α
μ (mm^−1^)	0.16
Crystal size (mm)	0.20 × 0.20 × 0.05

Data collection	
Diffractometer	Bruker APEXII CCD
Absorption correction	Multi-scan (*SADABS*; Bruker, 2014 ▸)
*T*_min_, *T*_max_	0.691, 0.746
No. of measured, independent and observed [*I* > 2σ(*I*)] reflections	29229, 6339, 4923
*R*_int_	0.038
(sin θ/λ)_max_ (Å^−1^)	0.661

Refinement	
*R*[*F* ^2^ > 2σ(*F* ^2^)], *wR*(*F* ^2^), *S*	0.041, 0.104, 1.02
No. of reflections	6339
No. of parameters	326
H-atom treatment	H-atom parameters constrained
Δρ_max_, Δρ_min_ (e Å^−3^)	0.42, −0.24

Computer programs: *APEX2* and *SAINT* (Bruker, 2014 ▸), *SHELXS* and *XP* (Sheldrick, 2008 ▸), *SHELXL* (Sheldrick, 2015 ▸), *Mercury* (Macrae *et al.*, 2020 ▸) and *publCIF* (Westrip, 2010 ▸).

## full crystallographic data

### Crystal data

**Table:**

C_26_H_34_O_4_Si_2_	*F*(000) = 1000
*M**_r_* = 466.71	*D*_x_ = 1.180 Mg m^−^^3^
Monoclinic, *P*2_1_/*c*	Mo *K*α radiation, λ = 0.71073 Å
*a* = 10.2628 (6) Å	Cell parameters from 8947 reflections
*b* = 20.5653 (13) Å	θ = 2.4–28.1°
*c* = 13.3923 (8) Å	µ = 0.16 mm^−^^1^
β = 111.6378 (15)°	*T* = 100 K
*V* = 2627.4 (3) Å^3^	Block, colourless
*Z* = 4	0.20 × 0.20 × 0.05 mm

### Data collection

**Table:**

Bruker APEXII CCD diffractometer	4923 reflections with *I* > 2σ(*I*)
ω– and φ–scans	*R*_int_ = 0.038
Absorption correction: multi-scan (*SADABS*; Bruker, 2014)	θ_max_ = 28.0°, θ_min_ = 2.4°
*T*_min_ = 0.691, *T*_max_ = 0.746	*h* = −13→13
29229 measured reflections	*k* = −27→27
6339 independent reflections	*l* = −17→17

### Refinement

**Table:**

Refinement on *F*^2^	Primary atom site location: structure-invariant direct methods
Least-squares matrix: full	Hydrogen site location: inferred from neighbouring sites
*R*[*F*^2^ > 2σ(*F*^2^)] = 0.041	H-atom parameters constrained
*wR*(*F*^2^) = 0.104	*w* = 1/[σ^2^(*F*_o_^2^) + (0.0483*P*)^2^ + 1.0291*P*] where *P* = (*F*_o_^2^ + 2*F*_c_^2^)/3
*S* = 1.02	(Δ/σ)_max_ = 0.001
6339 reflections	Δρ_max_ = 0.42 e Å^−^^3^
326 parameters	Δρ_min_ = −0.24 e Å^−^^3^
0 restraints	

### Special details

**Table:**

Geometry. All esds (except the esd in the dihedral angle between two l.s. planes) are estimated using the full covariance matrix. The cell esds are taken into account individually in the estimation of esds in distances, angles and torsion angles; correlations between esds in cell parameters are only used when they are defined by crystal symmetry. An approximate (isotropic) treatment of cell esds is used for estimating esds involving l.s. planes.

### Fractional atomic coordinates and isotropic or equivalent isotropic displacement parameters (Å^2^)

**Table:**

	*x*	*y*	*z*	*U*_iso_*/*U*_eq_	Occ. (<1)
Si1	0.20791 (5)	0.30679 (2)	0.57403 (3)	0.02248 (11)	
C1	0.7133 (2)	0.48696 (8)	0.37326 (13)	0.0302 (4)	
Si2	0.67617 (4)	0.34083 (2)	0.98331 (3)	0.01647 (10)	
O2	0.79449 (14)	0.51514 (6)	0.34281 (9)	0.0338 (3)	
C2	0.73267 (18)	0.46099 (7)	0.48068 (12)	0.0231 (3)	
O3	0.81087 (11)	0.36239 (6)	0.94866 (8)	0.0237 (2)	
C3	0.84777 (17)	0.46481 (7)	0.57425 (13)	0.0217 (3)	
H3	0.931227	0.485539	0.575825	0.026*	
O4	1.03876 (11)	0.42083 (5)	0.96159 (9)	0.0237 (2)	
C4	0.83931 (16)	0.43714 (7)	0.66845 (12)	0.0184 (3)	
C5	0.95276 (16)	0.44210 (7)	0.76950 (12)	0.0206 (3)	
H5	1.037576	0.462529	0.773525	0.025*	
C6	0.94047 (16)	0.41766 (7)	0.86068 (12)	0.0193 (3)	
C7	0.81345 (16)	0.38558 (7)	0.85467 (11)	0.0183 (3)	
C8	0.70463 (16)	0.37984 (7)	0.75858 (12)	0.0181 (3)	
H8	0.621620	0.358099	0.755637	0.022*	
C9	0.71283 (16)	0.40565 (7)	0.66304 (11)	0.0168 (3)	
C10	0.59432 (17)	0.40258 (7)	0.56348 (12)	0.0203 (3)	
C11	0.60720 (18)	0.43166 (8)	0.47435 (12)	0.0256 (4)	
C14	0.2766 (2)	0.38807 (11)	0.27074 (15)	0.0403 (5)	
H14A	0.228359	0.423527	0.286908	0.048*	0.793 (5)
H14B	0.225496	0.351833	0.231592	0.048*	0.793 (5)
H14C	0.299781	0.344558	0.260154	0.048*	0.207 (5)
H14D	0.181140	0.401044	0.247364	0.048*	0.207 (5)
C15	0.46697 (17)	0.37325 (8)	0.56158 (12)	0.0230 (3)	
C16	0.36373 (18)	0.34782 (8)	0.56664 (12)	0.0260 (4)	
C17	0.0501 (2)	0.34828 (10)	0.47995 (15)	0.0389 (5)	
H17A	0.048349	0.393362	0.503155	0.058*	
H17B	−0.034178	0.325613	0.479467	0.058*	
H17C	0.052507	0.347805	0.407497	0.058*	
C18	0.2142 (2)	0.22140 (9)	0.53083 (16)	0.0364 (4)	
H18A	0.214109	0.220951	0.457613	0.055*	
H18B	0.132165	0.197743	0.532449	0.055*	
H18C	0.299819	0.200466	0.579619	0.055*	
C19	0.21649 (19)	0.31364 (9)	0.71450 (13)	0.0318 (4)	
H19A	0.295737	0.288043	0.761874	0.048*	
H19B	0.129237	0.297148	0.719013	0.048*	
H19C	0.229034	0.359329	0.736794	0.048*	
C20	0.54271 (18)	0.40670 (8)	0.94724 (13)	0.0282 (4)	
H20A	0.584916	0.446626	0.985485	0.042*	
H20B	0.464002	0.393737	0.967605	0.042*	
H20C	0.508864	0.414466	0.869624	0.042*	
C21	0.59902 (19)	0.26318 (8)	0.91747 (13)	0.0282 (4)	
H21A	0.555877	0.269748	0.839637	0.042*	
H21B	0.527592	0.248566	0.944850	0.042*	
H21C	0.672816	0.230206	0.933128	0.042*	
C22	0.6523 (2)	0.31159 (11)	1.17885 (15)	0.0403 (5)	
H22A	0.606496	0.270853	1.146464	0.060*	
H22B	0.582055	0.346268	1.163324	0.060*	
H22C	0.697512	0.306043	1.256777	0.060*	
C23	0.76275 (17)	0.32974 (8)	1.13195 (12)	0.0214 (3)	
C24	0.87241 (19)	0.27524 (9)	1.15756 (14)	0.0316 (4)	
H24A	0.943282	0.286281	1.127394	0.047*	
H24B	0.826462	0.234372	1.126024	0.047*	
H24C	0.917511	0.270285	1.235607	0.047*	
C25	0.8359 (2)	0.39328 (9)	1.18330 (14)	0.0378 (5)	
H25A	0.878708	0.387841	1.261393	0.057*	
H25B	0.766840	0.428533	1.166339	0.057*	
H25C	0.908657	0.403970	1.154917	0.057*	
C26	1.16625 (17)	0.45412 (9)	0.97460 (14)	0.0316 (4)	
H26A	1.210789	0.433590	0.929264	0.047*	
H26B	1.229676	0.451859	1.049976	0.047*	
H26C	1.145690	0.499728	0.953463	0.047*	
O1	0.5726 (3)	0.47723 (11)	0.30613 (17)	0.0254 (5)	0.793 (5)
C12	0.4924 (3)	0.44639 (12)	0.36387 (16)	0.0206 (5)	0.793 (5)
H12	0.424850	0.478621	0.373199	0.025*	0.793 (5)
C13	0.4132 (2)	0.38915 (10)	0.30236 (14)	0.0227 (6)	0.793 (5)
H13	0.461811	0.353786	0.286346	0.027*	0.793 (5)
O1'	0.6138 (9)	0.4537 (4)	0.3060 (6)	0.0201 (16)	0.207 (5)
C12'	0.5382 (9)	0.4140 (4)	0.3596 (6)	0.018 (2)	0.207 (5)
H12'	0.543941	0.366360	0.346803	0.022*	0.207 (5)
C13'	0.3928 (10)	0.4372 (4)	0.3254 (6)	0.023 (2)	0.207 (5)
H13'	0.371872	0.480928	0.336729	0.028*	0.207 (5)

### Atomic displacement parameters (Å^2^)

**Table:**

	*U*^11^	*U*^22^	*U*^33^	*U*^12^	*U*^13^	*U*^23^
Si1	0.0213 (2)	0.0254 (2)	0.0191 (2)	−0.00752 (18)	0.00542 (17)	−0.00023 (17)
C1	0.0448 (11)	0.0291 (9)	0.0220 (8)	−0.0110 (8)	0.0187 (8)	−0.0028 (7)
Si2	0.0167 (2)	0.0190 (2)	0.01550 (19)	−0.00173 (16)	0.00805 (16)	−0.00036 (15)
O2	0.0491 (8)	0.0330 (7)	0.0283 (6)	−0.0114 (6)	0.0250 (6)	0.0016 (5)
C2	0.0345 (9)	0.0204 (8)	0.0206 (8)	−0.0043 (7)	0.0175 (7)	−0.0002 (6)
O3	0.0188 (6)	0.0348 (6)	0.0182 (5)	−0.0029 (5)	0.0077 (4)	0.0081 (5)
C3	0.0254 (8)	0.0190 (7)	0.0273 (8)	−0.0009 (6)	0.0176 (7)	0.0015 (6)
O4	0.0158 (5)	0.0290 (6)	0.0247 (6)	−0.0021 (4)	0.0058 (4)	0.0078 (5)
C4	0.0225 (8)	0.0158 (7)	0.0222 (7)	0.0009 (6)	0.0145 (6)	0.0011 (6)
C5	0.0173 (7)	0.0197 (7)	0.0280 (8)	0.0004 (6)	0.0119 (6)	0.0041 (6)
C6	0.0167 (7)	0.0185 (7)	0.0235 (7)	0.0031 (6)	0.0082 (6)	0.0036 (6)
C7	0.0199 (7)	0.0196 (7)	0.0189 (7)	0.0017 (6)	0.0113 (6)	0.0043 (6)
C8	0.0197 (7)	0.0185 (7)	0.0198 (7)	−0.0026 (6)	0.0116 (6)	0.0006 (6)
C9	0.0216 (8)	0.0144 (6)	0.0186 (7)	0.0004 (6)	0.0124 (6)	−0.0006 (5)
C10	0.0283 (8)	0.0183 (7)	0.0182 (7)	−0.0051 (6)	0.0129 (6)	−0.0022 (6)
C11	0.0351 (10)	0.0261 (8)	0.0175 (7)	−0.0088 (7)	0.0118 (7)	−0.0030 (6)
C14	0.0319 (10)	0.0516 (12)	0.0354 (10)	0.0004 (9)	0.0103 (8)	−0.0008 (9)
C15	0.0316 (9)	0.0249 (8)	0.0125 (7)	−0.0071 (7)	0.0081 (6)	−0.0014 (6)
C16	0.0310 (9)	0.0302 (9)	0.0160 (7)	−0.0090 (7)	0.0078 (7)	−0.0001 (6)
C17	0.0307 (10)	0.0473 (12)	0.0315 (10)	0.0019 (9)	0.0030 (8)	0.0040 (8)
C18	0.0412 (11)	0.0298 (9)	0.0421 (11)	−0.0103 (8)	0.0199 (9)	−0.0053 (8)
C19	0.0276 (9)	0.0456 (11)	0.0222 (8)	−0.0134 (8)	0.0092 (7)	0.0010 (7)
C20	0.0246 (9)	0.0318 (9)	0.0262 (8)	0.0051 (7)	0.0068 (7)	0.0001 (7)
C21	0.0359 (10)	0.0259 (8)	0.0241 (8)	−0.0076 (7)	0.0126 (7)	−0.0044 (7)
C22	0.0421 (11)	0.0612 (13)	0.0272 (9)	0.0061 (10)	0.0239 (9)	0.0098 (9)
C23	0.0260 (8)	0.0239 (8)	0.0164 (7)	0.0010 (6)	0.0104 (6)	0.0017 (6)
C24	0.0342 (10)	0.0336 (9)	0.0274 (9)	0.0094 (8)	0.0118 (8)	0.0089 (7)
C25	0.0518 (12)	0.0310 (10)	0.0190 (8)	−0.0006 (9)	−0.0004 (8)	−0.0029 (7)
C26	0.0177 (8)	0.0393 (10)	0.0333 (9)	−0.0071 (7)	0.0041 (7)	0.0079 (8)
O1	0.0336 (13)	0.0260 (11)	0.0198 (8)	−0.0034 (8)	0.0135 (8)	0.0048 (8)
C12	0.0278 (14)	0.0183 (11)	0.0186 (10)	0.0009 (10)	0.0119 (10)	0.0021 (8)
C13	0.0338 (13)	0.0223 (11)	0.0141 (9)	0.0017 (8)	0.0113 (9)	0.0005 (8)
O1'	0.023 (4)	0.029 (4)	0.016 (3)	−0.004 (3)	0.016 (3)	0.002 (3)
C12'	0.028 (5)	0.015 (4)	0.018 (4)	0.002 (3)	0.016 (3)	0.006 (3)
C13'	0.029 (5)	0.020 (4)	0.022 (4)	0.002 (3)	0.011 (4)	0.001 (3)

### Geometric parameters (Å, º)

**Table:**

Si1—C16	1.8427 (17)	C17—H17A	0.9800
Si1—C17	1.8533 (19)	C17—H17B	0.9800
Si1—C19	1.8559 (17)	C17—H17C	0.9800
Si1—C18	1.8575 (19)	C18—H18A	0.9800
C1—O2	1.203 (2)	C18—H18B	0.9800
C1—O1'	1.282 (8)	C18—H18C	0.9800
C1—O1	1.405 (3)	C19—H19A	0.9800
C1—C2	1.478 (2)	C19—H19B	0.9800
Si2—O3	1.6721 (11)	C19—H19C	0.9800
Si2—C21	1.8546 (17)	C20—H20A	0.9800
Si2—C20	1.8592 (17)	C20—H20B	0.9800
Si2—C23	1.8708 (15)	C20—H20C	0.9800
C2—C3	1.371 (2)	C21—H21A	0.9800
C2—C11	1.396 (2)	C21—H21B	0.9800
O3—C7	1.3554 (17)	C21—H21C	0.9800
C3—C4	1.415 (2)	C22—C23	1.531 (2)
C3—H3	0.9500	C22—H22A	0.9800
O4—C6	1.3574 (18)	C22—H22B	0.9800
O4—C26	1.4293 (19)	C22—H22C	0.9800
C4—C5	1.427 (2)	C23—C24	1.535 (2)
C4—C9	1.428 (2)	C23—C25	1.537 (2)
C5—C6	1.369 (2)	C24—H24A	0.9800
C5—H5	0.9500	C24—H24B	0.9800
C6—C7	1.437 (2)	C24—H24C	0.9800
C7—C8	1.363 (2)	C25—H25A	0.9800
C8—C9	1.4162 (19)	C25—H25B	0.9800
C8—H8	0.9500	C25—H25C	0.9800
C9—C10	1.437 (2)	C26—H26A	0.9800
C10—C11	1.384 (2)	C26—H26B	0.9800
C10—C15	1.431 (2)	C26—H26C	0.9800
C11—C12'	1.481 (8)	O1—C12	1.465 (3)
C11—C12	1.544 (3)	C12—C13	1.494 (3)
C14—C13	1.307 (3)	C12—H12	1.0000
C14—C13'	1.528 (9)	C13—H13	0.9500
C14—H14A	0.9500	O1'—C12'	1.481 (10)
C14—H14B	0.9500	C12'—C13'	1.470 (13)
C14—H14C	0.9500	C12'—H12'	1.0000
C14—H14D	0.9500	C13'—H13'	0.9500
C15—C16	1.206 (2)		
			
C16—Si1—C17	108.12 (9)	H18B—C18—H18C	109.5
C16—Si1—C19	107.58 (7)	Si1—C19—H19A	109.5
C17—Si1—C19	110.92 (9)	Si1—C19—H19B	109.5
C16—Si1—C18	106.58 (8)	H19A—C19—H19B	109.5
C17—Si1—C18	110.23 (9)	Si1—C19—H19C	109.5
C19—Si1—C18	113.17 (9)	H19A—C19—H19C	109.5
O2—C1—O1'	119.4 (3)	H19B—C19—H19C	109.5
O2—C1—O1	121.90 (16)	Si2—C20—H20A	109.5
O2—C1—C2	129.99 (17)	Si2—C20—H20B	109.5
O1'—C1—C2	106.1 (4)	H20A—C20—H20B	109.5
O1—C1—C2	108.01 (15)	Si2—C20—H20C	109.5
O3—Si2—C21	110.37 (7)	H20A—C20—H20C	109.5
O3—Si2—C20	109.88 (7)	H20B—C20—H20C	109.5
C21—Si2—C20	111.06 (8)	Si2—C21—H21A	109.5
O3—Si2—C23	102.17 (6)	Si2—C21—H21B	109.5
C21—Si2—C23	110.66 (7)	H21A—C21—H21B	109.5
C20—Si2—C23	112.38 (7)	Si2—C21—H21C	109.5
C3—C2—C11	123.00 (14)	H21A—C21—H21C	109.5
C3—C2—C1	129.09 (15)	H21B—C21—H21C	109.5
C11—C2—C1	107.88 (14)	C23—C22—H22A	109.5
C7—O3—Si2	130.71 (10)	C23—C22—H22B	109.5
C2—C3—C4	118.44 (14)	H22A—C22—H22B	109.5
C2—C3—H3	120.8	C23—C22—H22C	109.5
C4—C3—H3	120.8	H22A—C22—H22C	109.5
C6—O4—C26	117.07 (12)	H22B—C22—H22C	109.5
C3—C4—C5	121.33 (14)	C22—C23—C24	108.86 (14)
C3—C4—C9	119.35 (14)	C22—C23—C25	109.68 (15)
C5—C4—C9	119.28 (13)	C24—C23—C25	108.91 (15)
C6—C5—C4	120.57 (14)	C22—C23—Si2	109.46 (12)
C6—C5—H5	119.7	C24—C23—Si2	110.38 (11)
C4—C5—H5	119.7	C25—C23—Si2	109.53 (11)
O4—C6—C5	126.26 (14)	C23—C24—H24A	109.5
O4—C6—C7	113.81 (13)	C23—C24—H24B	109.5
C5—C6—C7	119.92 (14)	H24A—C24—H24B	109.5
O3—C7—C8	123.82 (13)	C23—C24—H24C	109.5
O3—C7—C6	115.95 (13)	H24A—C24—H24C	109.5
C8—C7—C6	120.23 (13)	H24B—C24—H24C	109.5
C7—C8—C9	121.31 (14)	C23—C25—H25A	109.5
C7—C8—H8	119.3	C23—C25—H25B	109.5
C9—C8—H8	119.3	H25A—C25—H25B	109.5
C8—C9—C4	118.67 (13)	C23—C25—H25C	109.5
C8—C9—C10	120.66 (13)	H25A—C25—H25C	109.5
C4—C9—C10	120.63 (13)	H25B—C25—H25C	109.5
C11—C10—C15	122.94 (14)	O4—C26—H26A	109.5
C11—C10—C9	117.63 (14)	O4—C26—H26B	109.5
C15—C10—C9	119.34 (13)	H26A—C26—H26B	109.5
C10—C11—C2	120.90 (15)	O4—C26—H26C	109.5
C10—C11—C12'	128.9 (3)	H26A—C26—H26C	109.5
C2—C11—C12'	104.3 (3)	H26B—C26—H26C	109.5
C10—C11—C12	129.02 (16)	C1—O1—C12	111.62 (18)
C2—C11—C12	109.51 (14)	O1—C12—C13	110.33 (17)
C13—C14—H14A	120.0	O1—C12—C11	102.26 (18)
C13—C14—H14B	120.0	C13—C12—C11	116.20 (18)
H14A—C14—H14B	120.0	O1—C12—H12	109.2
C13'—C14—H14C	120.0	C13—C12—H12	109.2
C13'—C14—H14D	120.0	C11—C12—H12	109.2
H14C—C14—H14D	120.0	C14—C13—C12	119.5 (2)
C16—C15—C10	175.98 (16)	C14—C13—H13	120.2
C15—C16—Si1	178.46 (16)	C12—C13—H13	120.2
Si1—C17—H17A	109.5	C1—O1'—C12'	112.2 (6)
Si1—C17—H17B	109.5	C13'—C12'—O1'	108.9 (7)
H17A—C17—H17B	109.5	C13'—C12'—C11	106.6 (7)
Si1—C17—H17C	109.5	O1'—C12'—C11	103.4 (6)
H17A—C17—H17C	109.5	C13'—C12'—H12'	112.5
H17B—C17—H17C	109.5	O1'—C12'—H12'	112.5
Si1—C18—H18A	109.5	C11—C12'—H12'	112.5
Si1—C18—H18B	109.5	C12'—C13'—C14	117.3 (6)
H18A—C18—H18B	109.5	C12'—C13'—H13'	121.4
Si1—C18—H18C	109.5	C14—C13'—H13'	121.4
H18A—C18—H18C	109.5		
